# Prediction of osteoporosis and osteopenia by routine computed tomography of the lumbar spine in different regions of interest

**DOI:** 10.1186/s13018-022-03348-2

**Published:** 2022-10-15

**Authors:** Guangyue Yang, Hansong Wang, Zhufeng Wu, Yinyu Shi, Yongfang Zhao

**Affiliations:** 1 Shi’s Center of Orthopedics and Traumatology, Shuguang Hospital Affiliated to Shanghai University of Traditional Chinese Medicine, Institute of Traumatology & Orthopedics, Shanghai Academy of Traditional Chinese Medicine, No. 528, Zhangheng Road, Shanghai, Pudong New Area 201203 China; 2 Sichuan Province Orthopaedic Hospital, No. 132, West Section 1, First Ring Road, Chengdu, Sichuan Province China

**Keywords:** Osteoporosis, Hounsfield unit, Bone mineral density (BMD), Dual X-ray absorptiometry (DXA), Opportunistic screening

## Abstract

**Background:**

We aimed to investigate the utility of Hounsfield units (HU) obtained from different regions of interest in opportunistic lumbar computed tomography (CT) to predict osteoporosis coupling with data of dual-energy X-ray absorptiometry (DXA).

**Methods:**

A total of 100 patients who attended a university hospital in Shanghai, China, and had undergone CT and DXA tests of the lumbar spine within 3 months were included in this retrospective review. Images were reviewed on axial sections, and regions of interest (ROI) markers were placed on the round, oval, anterior, left, and right of the L1–L4 vertebra to measure the HU. The mean values of CT HU were then compared to the bone mineral density (BMD) measured by DXA. Receiver operator characteristic curves were generated to determine the threshold for diagnosis and its sensitivity and specificity values.

**Results:**

The differences in CT HU of different ROI based on DXA definitions of osteoporosis, osteopenia, and normal individuals were statistically significant (*p* < 0.01). The HU values of the different ROI correlated well with the BMD values (Spearman coefficient all > 0.75, *p* < 0.01). The threshold for diagnosing osteoporosis varies from 87 to 111 HU in different ROIs, and the threshold for excluding osteoporosis or osteopenia is 99–125 HU.

**Conclusion:**

This is the first study on osteoporosis diagnosis of different ROI with routine CT lumbar scans. There is a strong correlation between CT HU of different ROI in the lumbar spine and BMD, and HU measurements can be used to predict osteoporosis.

## Background

Osteoporosis is a systemic skeletal disease characterized by reduced bone mass and destruction of the microstructure of bone tissue, leading to increased bone fragility and susceptibility to fracture, which seriously affects the quality of life of patients and can even be directly or indirectly life-threatening.

Fortunately, drugs such as Denosumab, Romosozumab, and Ibandronate are good at reducing fracture rates and increasing bone density [1, 2], so early diagnosis and treatment are essential to prevent fractures. Intervention is currently recommended in only 7–25% of screened women, well below 50% of fracture patients [3]. However, despite many efforts to diagnose osteoporosis early in high-risk groups, it is still underdiagnosed, many patients are only diagnosed at a late stage of fracture onset [4].

Dual-energy X-ray absorptiometry (DXA) is currently the gold standard recommended by the World Health Organization (WHO) for the diagnosis of osteoporosis, reflecting reduced bone mass and diagnosing osteoporosis [5]. Osteoporosis is an increasing public health concern with an aging population in Asia. The early diagnosis of patients at risk to prevent future fractures in the frail population represents a pivotal point in prevention [6]. Providing accurate, rapid, and convenient osteoporosis screening tools and techniques to enhance primary screening for osteoporosis and prevent the occurrence of fragility fractures is very important. At the same time, the widespread availability of conventional computed tomography (CT) imaging at all levels of hospitals and its widespread use in routine practice provide an opportunity to apply CT imaging for opportunistic screening of high-risk groups [7]. Lumbar spine bone density could be estimated indirectly by measuring Hounsfield units (HU) on CT of the abdomen or lumbar spine. In recent years, scholars have applied routine chest CT, and abdominal CT for opportunistic screening of osteoporosis in different populations in the USA, Europe, the Middle East, and Asia [8–10]. In these studies, BMD values obtained from DXA testing were compared with HU values obtained from random CT scans of the same patients, suggesting that HU values may identify patients with potentially low BMD. HU thresholds were calculated to differentiate between osteoporotic and non-osteoporotic (osteopenia or normal BMD), and normal and abnormal BMD (osteopenia or osteopenia).

This study analyzed and summarized the HU of different vertebrae and different regions of interest (ROI) of lumbar spine axial CT images to propose a more accurate and convenient screening method for osteoporosis.

## Methods

### Patients

This was a retrospective study, and cases were screened from patients who visited a university hospital in Shanghai from August 2019 to December 2021 and had both lumbar spine CT plain and DXA measurements within three months. All patients with a history of severe compression fracture or history of compression fracture surgery, oncological disease, hematological disease, known metabolic bone disease, or chronic kidney disease were excluded. Institutional review board approval was obtained for this retrospective study and written informed consent from patients was not required.

According to their T-Score values by WHO criteria, patients were classified as normal BMD, osteopenia, or osteoporosis. HU values of the lumbar spine (L1–L4) axial region of interest was measured retrospectively in this population. For each patient, HU values of the region of interest obtained by CT were compared with BMD values from DXA of the lumbar spine.

### DXA protocol and BMD measurement

We considered DXA as the gold standard for BMD assessment. DXA examinations of the lumbar spine and proximal hip were performed with the Lunar Prodigy (General Electric, medical system). BMD was measured at L1–L4 and femoral neck and total hip sites and expressed in g/cm^2^. According to the T-Scoring criteria, the BMD values derived from the DXA were classified as normal individuals, osteopenia, or osteoporosis.

### CT protocol and HU measurements

All CT scans were performed for diagnostic purposes due to various clinical indications, on a device (Philips Brilliance iCT). The CT parameters included slice thickness of 2.5 mm with 2.5 mm intervals, tube voltage of 120 kV, tube current of 250 mA, and scanning from L1–L4.

**Fig. 1:**
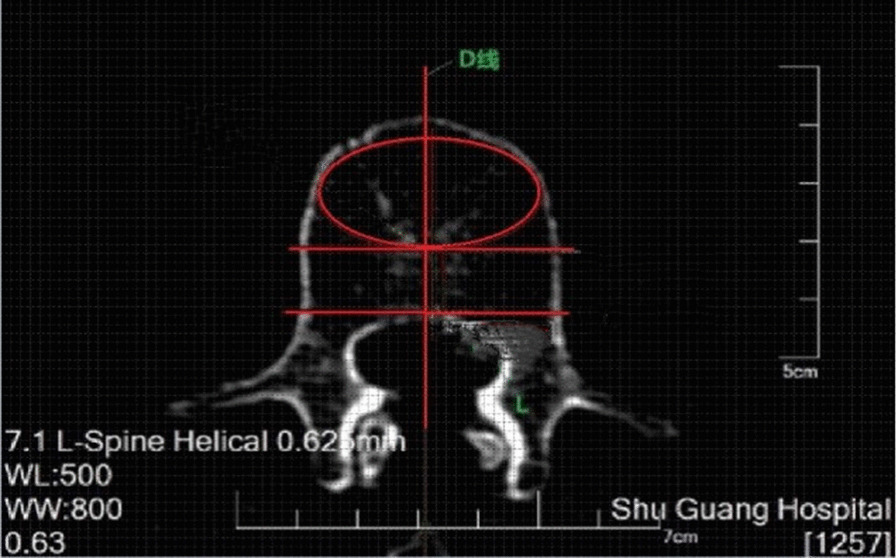
The oval ROI on the axial section at the center of the vertebral body

**Fig. 2:**
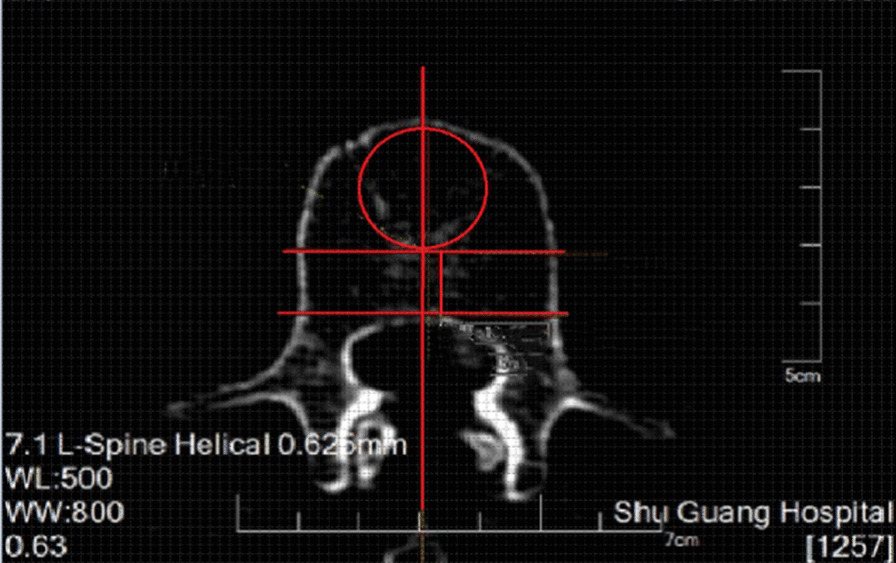
The round ROI on the axial section at the center of the vertebral body

**Fig. 3:**
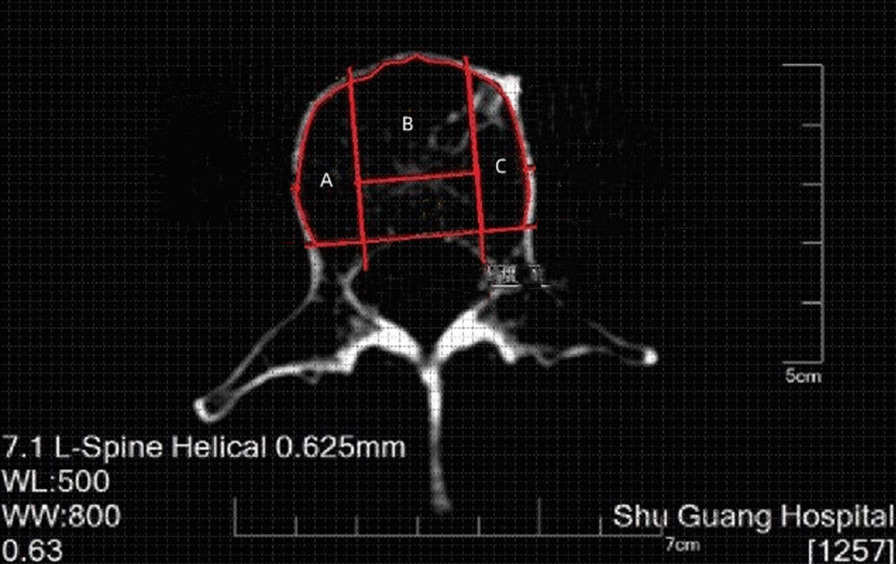
Division of right, front, and left ROI and calculation of HU values, in which zone A is the right ROI, zone B is the front ROI, and zone C is the left ROI

HU measurements were performed on a picture archiving and communication system (PACS, Neusoft software V5.5 version from Neusoft Group Corporation) using an axial section at the vertebral midline. The anterior 2/3 region of the vertebral body was identified and demarcated by measurement, followed by drawing the largest oval ROI in the anterior 2/3 region of the vertebral body (Fig. 1). The distance from the anterior 2/3 demarcation line of the vertebral body to the inner edge of the cortical bone was selected as the diameter for a round ROI (excluding the marginal cortical region) (Fig. 2). Using the midline of the longitudinal axis of the vertebral body as the symmetry axis, the anterior 2/3 region was selected for the anterior ROI, and the right ROI and left ROI were made according to the extension of the longitudinal border of the front ROI (Fig. 3). All the ROIs exclude the marginal cortical area. HU measurements were obtained for the L1–L4 vertebrae, for each ROI, independently by two experienced radiologists using PACS software, and the mean values were calculated and recorded.

### Statistics analysis

Statistical analyses were made using the software program PASW statistics 17.0 (SPSS Inc., Chicago, IL, USA). The test levels were *p* < 0.05 for statistically significant differences; *p* < 0.01 for highly statistically significant differences. To assess demographic baseline and HU values of each ROI region of CT, the Shapiro–Wilk test has been performed to investigate data distribution. For parametric data, mean and standard deviation were evaluated. The baseline comparability was assessed using analysis of variance (ANOVA), with *p* values > 0.1 considered satisfactory. For nonparametric data, median and interquartile were evaluated. The baseline comparability was assessed by the Kruskal–Wallis test, with *p* values > 0.1 considered satisfactory. Most HU values of different ROIs did not obey normal distribution, so they were expressed as median (M) and quartile spacing (Q1, Q3). A bivariate correlation test was applied: the correlation between HU values and DXA_BMD_ in each ROI region was analyzed separately, using Spearman's correlation test.

The diagnostic efficacy of the HU values in the different ROI regions of each vertebra was assessed by plotting the receiver operator characteristic (ROC) to obtain the area under the curve (AUC), with an AUC > 0.7 suggesting a favorable predictive value.

## Results

### Patients

A total of 100 subjects met the inclusion criteria, including 27 subjects with DXA_BMD_ diagnosis of osteoporosis, 40 subjects with osteopenia, and 33 subjects with normal BMD. Seventeen subjects were male and 83 subjects were female.

The subjects with osteoporosis were relatively older, followed by those with osteopenia and those with normal individuals were younger, with a significant difference in age distribution between the three groups (*p* < 0.01). The basic information about the subjects is shown in Table 1.

**Table 1:** Main Characteristics of the study population

Variable	DXA-based BMD category		
	Normal	Osteopenia	Osteoporosis
Gender			
*N* (Male)	12	4	1
*N* (Female)	21	36	26
Age (years)	55.00 (39.50,66.00)	66.00 (59.50,75.00)	69.00 (65.00,76.00)
BMI (kg/m^2^)	24.82 (21.52,27.22)	25.60 (22.80,27.47)	23.14 (20.78,26.06)
L1–L4 BMD (g/cm^2^)	1.24 (1.10,1.28)	0.99 (0.94,1.04)	0.77 (0.64,0.84)
Neck BMD (g/cm^2^)	0.91 (0.87,1.01)	0.74 (0.68,0.83)	0.65 (0.58,0.72)

### Differences in CT HU between osteoporosis, osteopenia, and normal in different ROI in the lumbar spine

Table 2 tabulates CT HU data of different ROIs stratified by the three DXA categorized bone statuses of normal, osteopenia, and osteoporosis. The HU values of each vertebral body of L1 to L4 were not significantly different, suggesting that the mean HU values of L1 to L4 vertebrae could represent the mean CT attenuation values of the lumbar spine. The results showed that there was a significant difference between the ROI-HU values of the three groups at different vertebral levels (all *p* < 0.001), specifically the normal > osteopenia > osteoporosis (Table 2).

**Table 2:** Tabulation of different ROI CT attenuation values, expressed in Hounsfield unit (HU) for L1 to L4 and their mean values and 95% confidence interval (CI), for DXA-defined normal, osteopenia, and osteoporosis subgroup

Vertebrae	Methods	Normal	Osteopenia	Osteoporosis	*p*
L1	BMD (g/cm^2^)	1.11 (1.02, 1.19)*	0.89 (0.84, 0.96)^▲^	0.71 (0.62, 0.77)^▲,^*	< 0.001
	Round ROI_CT_ (HU)	164.00 (131.00, 223.00)*	131.00 (75.25, 165.75)^▲^	73.00 (33.00, 91.00)^▲,^*	< 0.001
	Oval ROI_CT_ (HU)	172.00 (128.50, 219.00)*	128.00 (85.00, 165.00)^▲^	69.00 (36.00, 93.00)^▲,^*	< 0.001
	Right ROI_CT_ (HU)	163.00 (117.50, 196.00)*	116.50 (89.75, 148.25)^▲^	74.00 (50.00, 96.00)^▲,^*	< 0.001
	Front ROI_CT_ (HU)	169.00 (134.00, 219.50)*	135.00 (83.00, 168.25)^▲^	71.00 (38.00, 92.00)^▲,^*	< 0.001
	Left ROI_CT_ (HU)	189.00 (138.00, 203.00)*	130.00 (101.00, 163.50)^▲^	73.00 (47.00, 98.00)^▲,^*	< 0.001
L2	BMD (g/cm^2^)	1.18 (1.07, 1.27)*	0.94 (0.90, 0.98)^▲^	0.75 (0.63, 0.83)^▲,^*	< 0.001
	Round ROI_CT_ (HU)	163.00 (141.00, 213.00)*	106.00 (74.75, 139.25)^▲^	68.00 (38.00, 99.00)^▲,^*	< 0.001
	Oval ROI_CT_ (HU)	156.00 (127.00, 208.00)*	110.00 (73.50, 141.25)^▲^	73.00 (36.00, 106.00)^▲,^*	< 0.001
	Right ROI_CT_ (HU)	140.00 (109.50, 200.00)*	103.50 (91.25, 137.75)^▲^	71.00 (41.00, 102.00)^▲,^*	< 0.001
	Front ROI_CT_ (HU)	153.00 (127.50, 213.00)*	110.50 (80.00, 146.75)^▲^	69.00 (40.00, 104.00)^▲,^*	< 0.001
	Left ROI_CT_ (HU)	162.00 (126.00, 200.50)*	115.50 (84.25, 152.75)^▲^	71.00 (31.00, 105.00)^▲,^*	< 0.001
L3	BMD (g/cm^2^)	1.29 (1.15, 1.38)*	1.03 (0.95, 1.07)^▲^	0.80 (0.65, 0.86)^▲,^*	< 0.001
	Round ROI_CT_ (HU)	153.00 (111.50, 208.00)*	95.50 (61.00, 132.00)^▲^	53.00 (27.00, 75.00)^▲,^*	< 0.001
	Oval ROI_CT_ (HU)	152.00 (116.00, 205.00)*	94.00 (62.50, 138.50)^▲^	48.00 (28.00, 75.00)^▲,^*	< 0.001
	Right ROI_CT_ (HU)	154.00 (110.50, 199.00)*	100.50 (75.87, 118.50)^▲^	68.00 (36.00, 92.00)^▲,^*	< 0.001
	Front ROI_CT_ (HU)	152.00 (119.00, 210.00)*	95.00 (64.50, 135, 75)^▲^	54.00 (24.00, 79.00)^▲,^*	< 0.001
	Left ROI_CT_ (HU)	166.00 (134.00, 205.00)*	100.50 (78.50, 127.50)^▲^	58.00 (37.00, 91.00)^▲,^*	< 0.001
L4	BMD (g/cm^2^)	1.29 (1.15, 1.38)*	1.02 (0.95, 1.07)^▲^	0.82 (0.68, 0.88)^▲,^*	< 0.001
	Round ROI_CT_ (HU)	166.00 (118.00, 218.50)*	99.00 (60.50, 132.00)^▲^	58.00 (16.00, 81.00)^▲,^*	< 0.001
	Oval ROI_CT_ (HU)	164.00 (115.50, 206.50)*	94.00 (61.25, 130.50)^▲^	55.00 (23.00, 85.00)^▲,^*	< 0.001
	Right ROI_CT_ (HU)	179.00 (134.00, 247.00)*	134.00 (97.50, 173.25)^▲^	81.00 (46.00, 103.00)^▲,^*	< 0.001
	Front ROI_CT_ (HU)	171.00 (121.00, 222.00)*	101.50 (69.00, 135.25)^▲^	65.00 (22.00, 88.00)^▲,^*	< 0.001
	Left ROI_CT_ (HU)	196.00 (128.00, 226.50)*	119.00 (93.50, 152.50)^▲^	83.00 (53.00, 104.00)^▲,^*	< 0.001
L1–L4	BMD (g/cm^2^)	1.24 (1.10, 1.28)*	0.99 (0.92, 1.03)^▲^	0.77 (0.64, 0.84)^▲,^*	< 0.001
	Round ROI_CT_ (HU)	162.25 (123.25, 217.25)*	107.00 (68.88, 142.00)^▲^	53.00 (35.00, 85.25)^▲,^*	< 0.001
	Oval ROI_CT_ (HU)	158.75 (123.38, 213.13)*	102.63 (71.69, 140.81)^▲^	54.75 (32.25, 88.50)^▲,^*	< 0.001
	Right ROI_CT_ (HU)	156.75 (120.88, 206.88)*	111.50 (93.94, 149.13)^▲^	74.00 (47.25, 98.00)^▲,^*	< 0.001
	Front ROI_CT_ (HU)	159.00 (125.13, 214.75)*	107.25 (70.94, 144.75)^▲^	57.25 (53.50, 85.75)^▲,^*	< 0.001
	Left ROI_CT_ (HU)	171.25 (135.75, 214.50)*	115.00 (96.63, 142.50)^▲^	68.00 (41.75, 103.75)^▲,^*	< 0.001

^▲^Indicates a statistically significant difference compared to the normal

*Indicates a statistically significant difference compared to osteopenia

We also compared the HU values in different ROIs, the left ROI had the highest HU value (116.38 (77.81, 165.25)), and the oval ROI had the lowest HU value (105.00 (68.06, 157.44)), but there was no statistically significant difference between the different ROIs (Kruskal Wallis test, *H* = 2.81, *P* = 0.59).

### Correlation between lumbar vertebral CT HU and DXABMD in different regions of interest

**Fig. 4:**
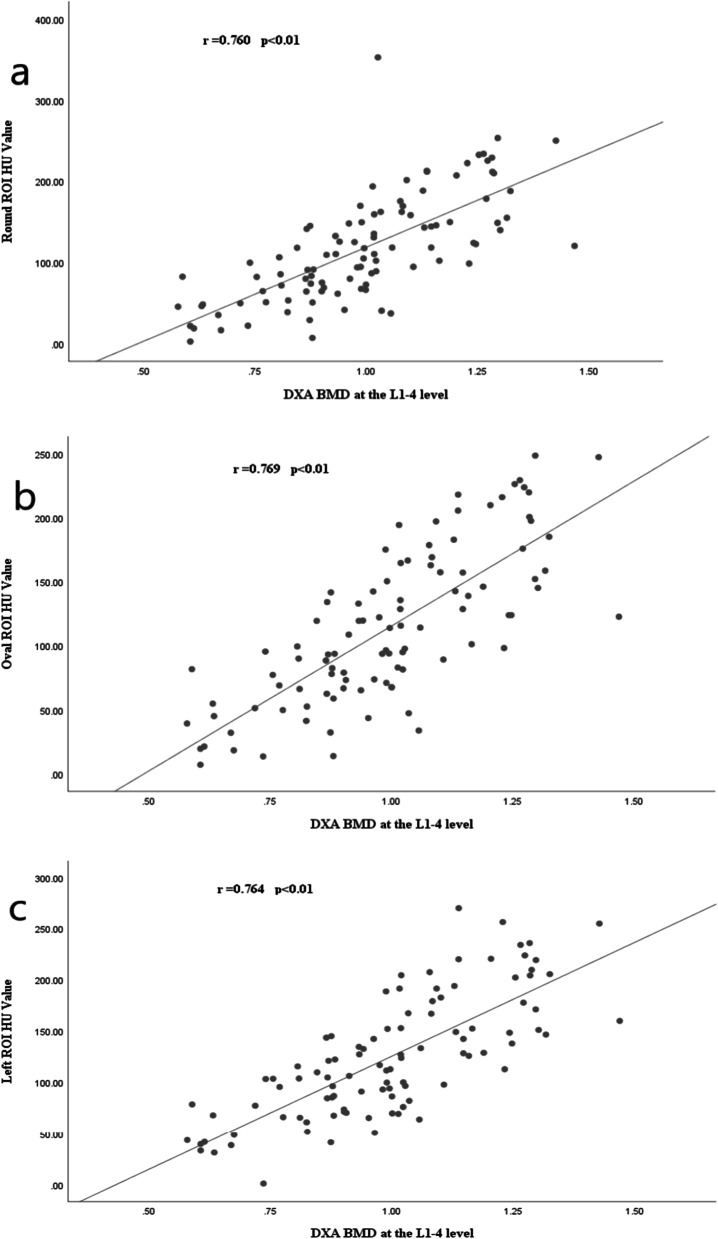

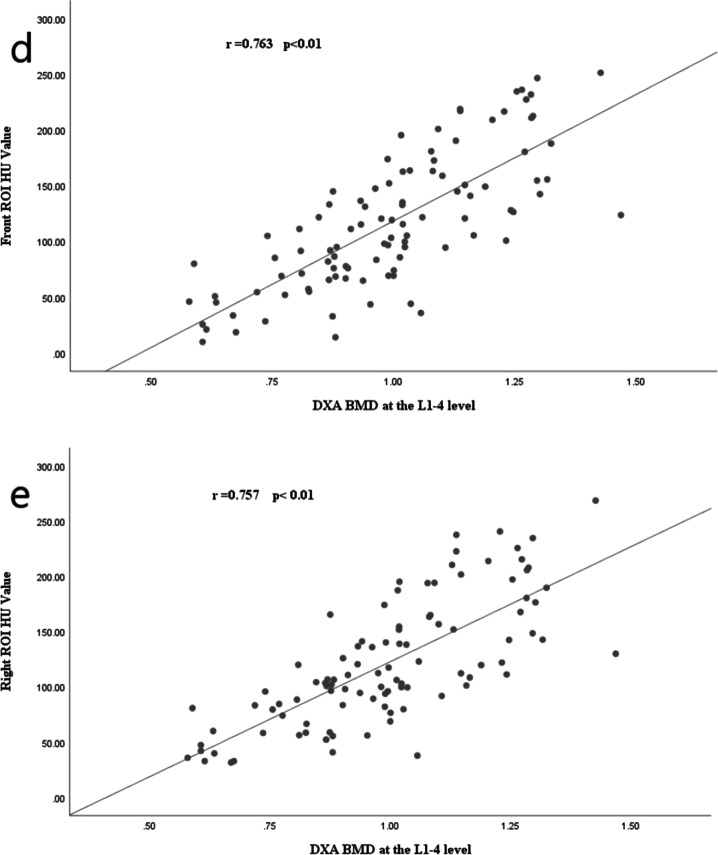
Scatter plots showing the strongest correlations between the HU values of different ROI and BMD at the L1–L4 level (all r > 0.75, *p* < 0.01): **a** round ROI, **b** oval ROI, **c** left ROI, **d** front ROI, **e** right ROI

Correlations between round, oval, right, anterior column, and left ROI-CT HU and DXA_BMD_ were compared separately at the L1–L4 level and showed good correlations (all correlation coefficients *r* > 0.75, all *p* < 0.01), as detailed in Fig. 4a–e.

### Cutoff HU values and ROC curves of different ROI

We devised optimal cutoff HU values using the mean values of L1 to L4 HU to maximize the sensitivity and specificity for the exclusion and diagnosis of osteoporosis using the Youden index in the ROC curve analysis. The threshold for diagnosing osteoporosis varies from 87 to 111 HU in different regions of interest, and the threshold for excluding osteoporosis or osteopenia is 99–125 HU. Diagnostic performance of mean HU cutoff values in different ROI for distinguishing normal from low BMD and for distinguishing osteoporosis from nonosteoporosis are shown in Table 3.

**Table 3:** Diagnostic performance of mean CT attenuation cutoff values in different ROI, expressed in Hounsfield unit (HU), for distinguishing normal from low bone mineral density and for distinguishing osteoporosis from nonosteoporosis

Parameters	Osteoporosis				Exclusion of osteoporosis or osteopenia			
	Cutoff HU	Sensitivity (%)	Specificity (%)	Jorden Index	Cutoff HU	Sensitivity (%)	Specificity (%)	Jorden Index
Round ROI	87.75	79.5	81.5	0.61	118.13	84.8	73.1	0.58
Oval ROI	91.63	76.7	81.5	0.58	97.13	90.9	76.1	0.55
Right ROI	106.38	69.9	92.6	0.62	107.63	84.8	65.7	0.51
Left ROI	110.88	69.9	88.9	0.56	125.00	88.9	89.1	0.78
Front ROI	91.75	79.5	81.5	0.61	121.25	81.8	74.6	0.56

**Fig. 5:**
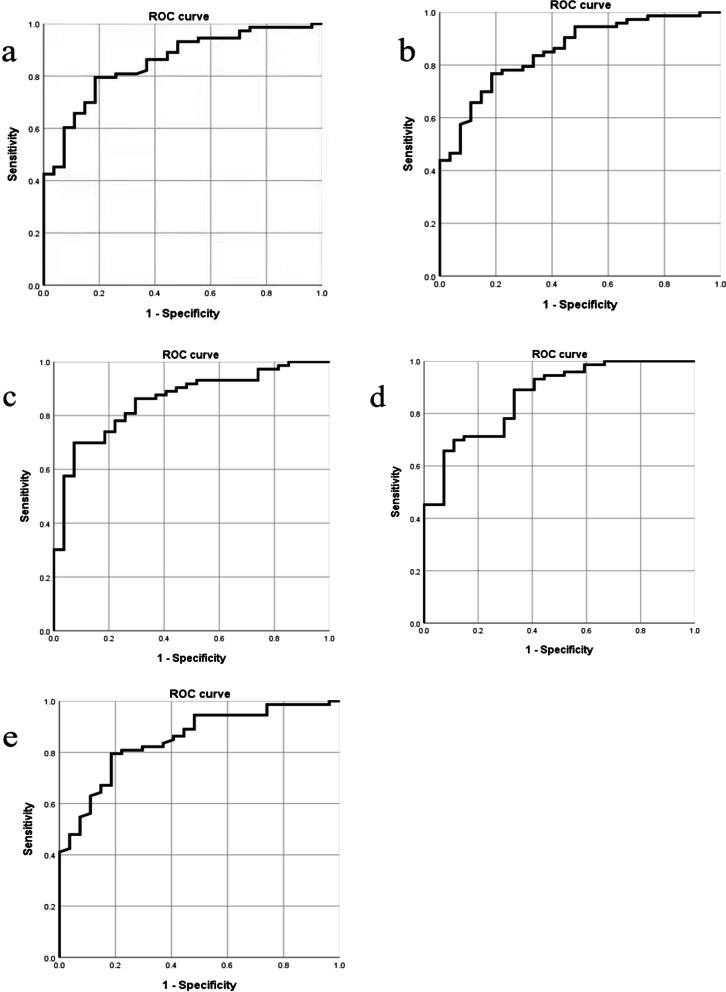
ROC curve for predicting osteoporosis based on HU measurement compared with DXA scans: **a** round ROI, **b** oval ROI, **c** left ROI, **d** right ROI, **e** front ROI

In round ROI, a mean HU threshold of ≤ 87.75 was 79.5% sensitivity and 81.5% specificity for distinguishing osteoporosis from nonosteoporosis with the AUC of the ROC curve as 0.852 (95% CI 0.775–0.93) (Fig. 5a). In oval ROI, a mean HU threshold of ≤ 91.63 was 76.7% sensitivity and 81.5% specificity for distinguishing osteoporosis from nonosteoporosis with the AUC of ROC curve as 0.853 (95% CI 0.776–0.930) (Fig. 5b). In the right ROI, a mean HU threshold of ≤ 106.38 was 69.9% sensitivity and 85.7% specificity for distinguishing osteoporosis from nonosteoporosis with the AUC of the ROC curve as 0.857 (95% CI 0.78–0.934) (Fig. 5c). In left ROI, a mean HU threshold of ≤ 110.88 was 69.9% sensitivity and 88.9% specificity for distinguishing osteoporosis from nonosteoporosis with the AUC of ROC curve as 0.936 (95% CI 0.891–0.982) (Fig. 5d). In front ROI, a mean HU threshold of ≤ 91.75 was 79.5% sensitivity and 81.5% specificity for distinguishing osteoporosis from nonosteoporosis with the AUC of ROC curve as 0.85 (95% CI 0.771–0.928) (Fig. 5e).

## Discussion

Our study investigated the possibility of using HU values obtained from random CT scans of the lumbar spine to predict abnormal BMD and to establish a threshold of HU values for determining osteoporosis. And we did a correlation analysis between CT values and DXA BMD for different regions of interest in the lumbar spine. We found a significant correlation between the DXA-derived BMD and the lumbar spine HU values revealed by CT of the lumbar spine.

In recent years, the use of HU values on CT images to quantitatively assess local bone quality has become more frequent. Lee et al. [11] compared the correlation between lumbar vertebral HU values and DXA on CT images and demonstrated that transverse and sagittal HU measurements were consistent with each other. To further validate the screening method for opportunistic osteoporosis, enhanced CT images of the abdomen were compared with CT plain images of the proximal femur and showed a nonsignificant difference. Thus, their data suggest that both non-enhanced and contrast-enhanced images can be used for screening for opportunistic osteoporosis with similar outcomes [12]. Otherwise, HU values of the lumbar spine can be used as a complementary method to identify undiagnosed spinal osteoporosis in patients with lumbar degenerative diseases [13]. Some studies have shown a clear correlation between HU and BMD [14]. Schreiber et al. [15] also found a significant positive correlation between HU and BMD (Pearson coefficient of 0.44). A study of the Hong Kong region showed the correlation between the mean HU values with DXA BMD of Pearson coefficient of 0.62 [8]. Another study of the Middle Eastern population showed the correlation between DXA-derived BMD T-scores and HU values measured on CT scans (*r* = 0.526). In our study, HU values for each ROI were strongly correlated with BMD (all correlation coefficients *r* > 0.75, *p* < 0.01).

As for the selection of ROIs, different research teams chose different regions of interest, in summary, specifically elliptical ROI regions in the sagittal position [9], elliptical ROI in cross section [16], round ROIs [17], etc. Of course, either ROI selection method emphasizes attention to avoiding osteophytes, spinal nerves, posterior venous plexus, local heterogeneity, or any image-related artifacts [18] and is more reflective of bone quality than a simple planar measurement of DXA BMD. This simple method does not require body casts, angulation along the disk plane, or consideration of multiple levels of assessment or muscle and fat location [19, 20]. In this study, we choose oval ROI, round ROI, left ROI, right ROI, and front ROI on axial images, and we also avoided spinal nerves, posterior venous plexus, and image-related artifacts. Our study showed that the oval ROI had the lowest HU value, though there was no statistically significant difference between them. Considering the lower HU values are more correlated with osteoporosis and the ease of manipulation in drawing ROIs, round or oval ROI can be the ideal region.

Using conventional CT for opportunistic screening for osteoporosis, scholars prefer to use a separate L1 vertebra, stemming from the fact that chest CT and abdominal CT can be involved. However, we believe that increasing the number of vertebrae measured may improve accuracy and precision, as it facilitates the use of HU data when one or more vertebrae are technically unsuitable for measurement. To determine more precisely and carefully the sensitivity of changes in vertebral ROI on CT of the lumbar spine in patients at high risk of fracture, the L1–L4 vertebrae were chosen for this study.

Samuel Jang et al.[9] showed that, at a population level, L1 trabecular CT HU was associated with an age-related BMD loss of 2.5 HU per year on average. large cohort studies (*n* = 1867) reported that a diagnostic cut point of 110 HU was more than 90% specific for osteoporosis [7]. Other studies with smaller sample sizes have shown HU thresholds of 136 HU [21] and 99 HU [22] for osteoporosis. In general, lower L1 HU values, such as 90–110 HU, are more specific and less sensitive for osteoporosis, while higher HU thresholds are less specific and more sensitive. A study of patients over 65 years of age with a follow-up interval of 5.8 years suggested that L1 trabecular attenuation of 90 HU or less may indicate a higher risk of fracture [23]. The current study had an osteoporosis threshold of 84–111 HU. However, the design of this study did not include a subject-specific FRAX (Fracture Risk Assessment Tool) score and long-term follow-up, so we would prefer to carry out a determination and assessment of the subject's fracture risk in future studies.

There are some limitations to this study, firstly our study design was retrospective and future prospective studies are necessary to consolidate and optimize our results. Secondly, we did not assess the relationship between ROI-CT HU and fracture risk. Thirdly, although a standardized procedure has been established, the specific operations are still performed by radiologists and the labor cost is high if a large number of opportunistic screenings in high-risk groups need to be completed.

## Conclusion

Our study is, to our knowledge, the first study on osteoporosis diagnosis of different ROI with routine CT lumbar scans. There is a strong correlation between CT HU of different ROI in the lumbar spine and BMD, and HU measurements can be used to predict osteoporosis. Opportunistic screening for osteoporosis and osteopenia by routine computed tomography scans should be increasingly used in routine practice.

## Data Availability

The datasets used and analyzed during the current study are available from the corresponding author upon reasonable request.

## Abbreviations

HU: Hounsfield units
CT: Computed tomography
DXA: Dual-energy X-ray absorptiometry
ROI: Regions of interest
BMD: Bone mineral density
ROC: Receiver operator characteristic
WHO: World Health Organization
AUC: Obtain the area under the curve
FRAX: Fracture Risk Assessment Tool
